# Correction: Political participation among deaf youth in Great Britain

**DOI:** 10.1371/journal.pone.0333019

**Published:** 2025-09-19

**Authors:** Francisco Espinoza, Alys Young, Claire Dodds

Figs 1 and 2 were uploaded incorrectly. Please see the correct Figs 1 and 2 here.

**Fig 1:**
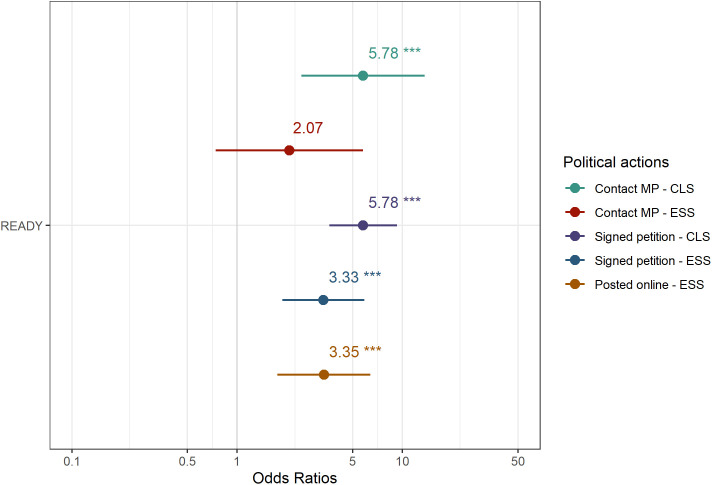
Differences in the probability of engagement in political participation (odds ratio). The figure presents the odds ratio of involvement for READY participants in signing petitions, posting online and contacting their MP, compared to the probabilities among the general population.

**Fig 2:**
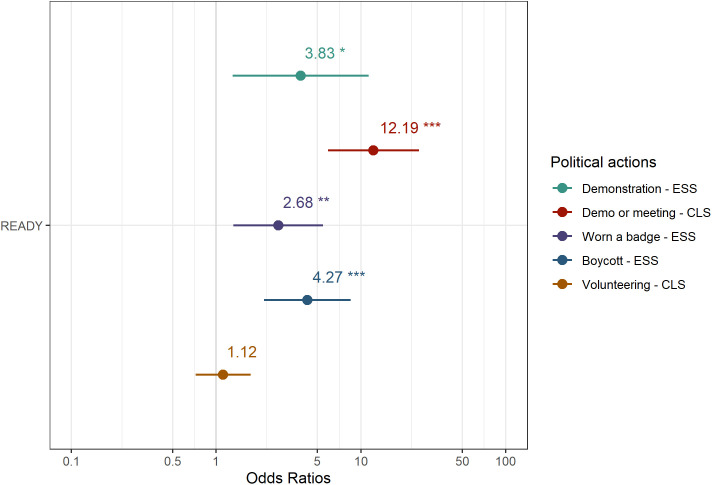
Differences in the probability of engagement in political participation (odds ratio). The figure presents the odds ratio of involvement for READY participants in attending demonstrations, political meetings, wearing a badge, boycotting a product and volunteering, compared to the probabilities among the general population.
