# Supplementary material for: Political participation among deaf youth in Great Britain
Source: PLoS One. 2024 Apr 4;19(4):e0301053. doi: 10.1371/journal.pone.0301053 (PMC10994419; doi:10.1371/journal.pone.0301053)
Supplement: Attachment — Submitted filename: Response to reviewers.docx [file pone.0301053.s001.docx]

Manchester, January 2024

Dear Dr Al-Yateem,

Academic Editor PLOS ONE

Thank you for your letter and the opportunity to submit a revised version of our manuscript. We also appreciate the reviewers’ comments, which will surely help us to enhance the manuscript's clarity.

Please find our point-by-point response to them and the journal requirements below.

Section 1. Journal Requirements:

1. When submitting your revision, we need you to address these additional requirements.

Please ensure that your manuscript meets PLOS ONE's style requirements, including those for file naming. The PLOS ONE style templates can be found at 
<https://journals.plos.org/plosone/s/file?id=wjVg/PLOSOne_formatting_sample_main_body.pdf> and 
<https://journals.plos.org/plosone/s/file?id=ba62/PLOSOne_formatting_sample_title_authors_affiliations.pdf>

RESPONSE: We have ensured that the style and file naming conventions have complied with PLOS ONE requirements. We have ensured that we are using the most updated PLOS ONE template for LaTeX (v3.6, August 2022), and thus our file should match with the journal standards.

2.  Did you know that depositing data in a repository is associated with up to a 25% citation advantage (<https://doi.org/10.1371/journal.pone.0230416>)? If you’ve not already done so, consider depositing your raw data in a repository to ensure your work is read, appreciated and cited by the largest possible audience. You’ll also earn an Accessible Data icon on your published paper if you deposit your data in any participating repository (<https://plos.org/open-science/open-data/#accessible-data>).

RESPONSE: Our main dataset is already a repository already in accordance with our funder’s requirements and it is under embargo, but we have created a smaller anonymized version with the purpose of replication of the analysis developed in this research article. The data is available in Figshare and can be accessed using following the DOI 10.48420/24968175

3. Thank you for stating the following financial disclosure: 
The READY study is funded by the National Deaf Children’s Society (UK). The views expressed in this article are those of the authors and not necessarily those of NDCS.
 
Please state what role the funders took in the study.  If the funders had no role, please state: "The funders had no role in study design, data collection and analysis, decision to publish, or preparation of the manuscript." 
If this statement is not correct you must amend it as needed. 
Please include this amended Role of Funder statement in your cover letter; we will change the online submission form on your behalf.

RESPONSE: We can confirm the following: "The funders had no role in study design, data collection and analysis, decision to publish, or preparation of the manuscript." We have included this amended Role of Funder statement in the cover letter.

4. Thank you for stating the following in the Acknowledgments Section of your manuscript: 
The READY study is funded by the National Deaf Children’s Society (UK). The views expressed in this article are those of the authors and not necessarily those of NDCS. The full READY research team also includes Dr Garry Squires, Dr Katherine Rogers and Dr Helen Chilton who are co-investigators on this research project. We sincerely thank all deaf young people who took the time to respond to our survey and provide data on their lives.

We note that you have provided funding information that is not currently declared in your Funding Statement. However, funding information should not appear in the Acknowledgments section or other areas of your manuscript. We will only publish funding information present in the Funding Statement section of the online submission form. 
Please remove any funding-related text from the manuscript and let us know how you would like to update your Funding Statement. Currently, your Funding Statement reads as follows: 
The READY study is funded by the National Deaf Children’s Society (UK). The views expressed in this article are those of the authors and not necessarily those of NDCS.

Please include your amended statements within your cover letter; we will change the online submission form on your behalf.

RESPONSE: We have removed the funding statement in the acknowledgements section and as per point 3 above changed it in line with journal requirements in the online submission form, and the cover letter as explained in Point 3. We have retained the acknowledgement of the other members of the READY study team in the acknowledgement section.

5. We note that you have indicated that there are restrictions to data sharing for this study. For studies involving human research participant data or other sensitive data, we encourage authors to share de-identified or anonymized data. However, when data cannot be publicly shared for ethical reasons, we allow authors to make their data sets available upon request. For information on unacceptable data access restrictions, please see <http://journals.plos.org/plosone/s/data-availability#loc-unacceptable-data-access-restrictions>.

Before we proceed with your manuscript, please address the following prompts:

a) If there are ethical or legal restrictions on sharing a de-identified data set, please explain them in detail (e.g., data contain potentially identifying or sensitive patient information, data are owned by a third-party organization, etc.) and who has imposed them (e.g., a Research Ethics Committee or Institutional Review Board, etc.). Please also provide contact information for a data access committee, ethics committee, or other institutional body to which data requests may be sent.

b) If there are no restrictions, please upload the minimal anonymized data set necessary to replicate your study findings to a stable, public repository and provide us with the relevant URLs, DOIs, or accession numbers. Please see <http://www.bmj.com/content/340/bmj.c181.long> for guidelines on how to de-identify and prepare clinical data for publication. For a list of recommended repositories, please see <https://journals.plos.org/plosone/s/recommended-repositories>. You also have the option of uploading the data as Supporting Information files, but we would recommend depositing data directly to a data repository if possible.

Please update your Data Availability statement in the submission form accordingly.

RESPONSE: There are no ethical or legal restrictions on sharing the de-identified data set. Following the restrictions associated with the time embargo on the whole data set, we have created a specific version for replication analysis. The data set and the script syntax used in the analysis were uploaded to the public depository Figshare, and can be accessed using the DOI 10.48420/24968175. We have updated the Data Availability statement in the submission form to reflect this.

Section 2. Comments from reviewer 1

Topic: The article is developed around a very pertinent topic for different research fields as sociology, youth studies, education and inclusion: political participation of deaf young people. Through a quantitative approach the study aims to compare not deaf and deaf young people levels of political participation. Less attention has been given to policital participation among young people from the deaf culture.

Abstract: The abstract includes information regarding the research gap, method, results and recommendations, but would benefit from a clear theoretical and conceptual standpoint underlying the research and data analysis.

RESPONSE: The reviewer is correct that we did not treat young people from deaf culture as a distinct group because we were problematizing the extent of affiliation that this generation of young deaf people had with deaf culture, rather than perceiving it as a binary distinction. We have strengthened the abstract to clearly show the conceptualisation of deaf youth as neither disabled nor culturally deaf, but that the fluidity of their affiliations in terms of identity and contact with other deaf people was itself of potential influence in the diversity of their political participation and in relation to our disability and general population comparators.

Introduction: the introductions starts by providing an analytical context for youth participation, in particular recent forms of participation or involvement in social movements and the absence ok knowledge regarding how young deaf are part of those.
Although the concept of interseccionality is mentioned it seems that deafness is the main characteristic (variable) that interfere in being involved in practices of participation and it sounds that the author is pointing to a more homogenous perspective of Deaf community.
Moreover, the concept of political participation needed to be defined and clarified at this point of the article as in the end of the introduction some results and claims are presented but without any clear guidance regarding the main conceptual organisers.

RESPONSE: It was not our intention to present a homogeneous view of the deaf community or deaf people more broadly – in fact, the opposite. Therefore, it is helpful that the reviewer seems to suggest we have done this to enable us to clarify further. We have done so in the text and with further references. We have defined and clarified the concept of political participation we are using here in the introduction.

Literature Review: This section focus on contribution of the literature to understand political participation of people with disabilities and raise relevant questions regarding levels of participation among that population and discusses specifically how participation among deaf people. However, I suggest to further discuss some aspects that sounds incomplete. For example, the sentence in page 3 sound incomplete, our at least needed some additional information: “In contrast, the literature on Deaf people who are sign language users and political 75 participation is distinct”. Could you elaborate a bit more on this idea.

RESPONSE: We have changed the paragraphing to show more clearly the relationship between our statement on the distinct nature of deaf cultural political participation campaigns and the examples given in what had previously been the next paragraph. We have also added some further clarifying text.

[Literature review] This section needs some review at two levels: level of structure as it is a bit circular and moving back and forward around the issue of disables and Deaf youth, mixing political rights and citizenship discussion with identity deriving sign language. Although it is understandable the reference to all of this subjects the section needs a clear sequence of topics to which are given relevance by the main aim of this article. This leads to the second level: content level. To include literature review regarding political participation of young people in general sounds necessary, not only because implicitly it is against those theoretical perspectives that Deaf youth participation is understood, but also because this study aims to compare Deaf youth with youth in general. The debate on political participation and what it is included in that definition seems necessary in this context. Suggestion: I suggest to include the last paragraph related to READY either in the introduction or in the Methods section.

RESPONSE: We have previously incorporated information on political participation among youth in the general population within the literature review section. However, we recognize the need for a more explicit explanation of the specific elements included in the data related to political participation for the clarity of this article. To address this, we have reorganized part of the literature review. We have also enhanced the transitions between these various bodies of literature, aiming to establish more explicit connections between them.

We note that the reviewer has used the capitalised D in reference to their comments on Deaf youth. However, we have been clear in our distinction between deaf youth in general and only using the capitalised form when relating clearly and firmly to those who are members of Deaf culture and claim a Deaf identity based on sign language and culture. This is distinct from other forms of deaf identity that may not be on the grounds of sign language and Deaf culture, which we discuss in the paragraph beginning ‘group identity’. Therefore, we would suggest that some of the issues the reviewer raises perhaps arise from that difference in their point of view with our usage of the terms deaf youth and Deaf youth. Nonetheless, we have reiterated through some additional material that the sign language arguments concerning culturally Deaf people are synonymous with claims to citizenship and political rights as these are denied through lack of recognition of the status of signed languages and legal rights in many countries.

About moving the last paragraph related to READY to the methods section, we followed the journal guidelines that stated that the research questions should follow on at the end of the literature review section and to make those clear we required this background paragraph concerning the READY study. We pick up on further details of that study later on in the methods section. We would therefore prefer to keep this paragraph where it is currently located in the literature review.

Methods: Methodological procedures, as well as specifically Ethical procedures are clear and presented in detail. Procedures regarding how hypothesis were approached are detailed and well explained, supported by similar actions taken before. Regarding the third hypothesis, the PCA resulted in three factors with consistency and with alpha values that are good for social sciences. One factor has only one item and this would deserve a clear justification. Moreover, theoretical support of the 3 factors would be beneficial for clarity and pertinence.

RESPONSE: We have added clarity in this section. Our definitions of the dimensions are based on previous definitions by empirical research, which we have adapted following the analysis of internal consistency.

Results and discussion: The descriptive statistics could provide information to understand results intra sample (READY respondents) to understand if deafness is the main variable to explain different results. This aspect is more clear in the following sections presenting interesting results, but the gender differences found, for example, are not clearly explained and discussed. This section is mainly presented at the descriptive level. I suggest proposing an explanatory framework to support results and to connect those with theory. There are signs that the Deaf young people are diverse intra sample and this is not completely explored.

RESPONSE: The discussion section has been enhanced by linking our results back with the main theoretical expectations and existing bibliography. We have provided further comments on the gender differences and connected them with previously cited research covering differences in the general population. We have strengthened the role of the explanatory hypothesis presented and linked back to the theoretical explanations presented.

What is the contribution of this study to advance new layers of understanding regarding youth participation? In the abstract the last sentence points to some contributions to policies development, but the article either in results or in the conclusion does not elaborate on this.

RESPONSE: We have highlighted the contribution of our research in the discussion and then in the conclusion sections. We have made sure to revisit the theoretical debates presented in the literature review section to provide a clearer connection with our findings. We have also made explicit our contributions in the conclusion section.

Section 3. Comments Reviewer 2

An excellent manuscript to be published which compares the levels of political participation among deaf youth with those of their peers in the general population and discusses how sociodemographic factors, self-identification as deaf, and meaningful interactions with other deaf people contribute to their political engagement. However, this manuscript needs more discussion, which can be improved by referring to the highlighted literature review.

RESPONSE: We have augmented the engagement with previously cited literature, including now more explicit references in the discussion section and the conclusions of the manuscript. In the first, we connect our hypothesis back to the main theory and to the empirical findings of previous research. In the conclusions, we have also debated more broadly on the implications of the paper, the connections with previous findings and specific contributions of the article.

We extend our sincere appreciation to the reviewers for generously dedicating their time and expertise to evaluating our article. We are genuinely grateful for the insightful comments provided. After thorough consideration, we have implemented the suggested modifications and believe that we have met the requested criteria.

We look forward to receiving updates.

Best regards,

Francisco Espinoza
